# ROS-Based Navigation and Obstacle Avoidance: A Study of Architectures, Methods, and Trends

**DOI:** 10.3390/s25144306

**Published:** 2025-07-10

**Authors:** Zhe Wei, Sen Wang, Kangyelin Chen, Fang Wang

**Affiliations:** 1 School of Computer Science, Civil Aviation Flight University of China, Guanghan 618307, China; chengnan@cafuc.edu.cn; 2 School of Science, Civil Aviation Flight University of China, Guanghan 618307, China; 15517966631@163.com (K.C.); cafuc_wf@foxmail.com (F.W.)

**Keywords:** ROS navigation stack, autonomous navigation, obstacle avoidance, path planning, sensor fusion

## Abstract

With the widespread adoption of the Robot Operating System (ROS), technologies for autonomous navigation in mobile robots have advanced considerably. ROS provides a modular navigation stack that integrates essential components, such as SLAM, localisation, global path planning, and obstacle avoidance, forming the foundation for applications including service robotics and autonomous driving. Nonetheless, achieving safe and reliable navigation in complex and dynamic environments remains a formidable challenge, due to the need for real-time perception of moving obstacles, sensor fusion requirements, and the demand for robust and efficient algorithms. This study presents a systematic examination of the ROS-based navigation stack and obstacle-avoidance mechanisms. The architecture and implementation principles of the core modules are analysed, along with a comparison of the features and application suitability of common local planners such as the Dynamic Window Approach (DWA) and Timed Elastic Band (TEB). The key technical challenges in autonomous navigation are summarised, and recent advancements are reviewed to outline emerging trends in ROS-based systems, including integration with deep learning, multi-robot coordination, and real-time optimisation. The findings contribute to a deeper theoretical understanding of robotic navigation and offer practical guidance for the design and development of autonomous systems.

## 1. Introduction

With the continuous advancement of Artificial Intelligence, embedded sensing, and intelligent control technologies, Autonomous Mobile Robots (AMRs) are being increasingly deployed across a range of domains, including industrial manufacturing, smart logistics, urban services, and healthcare assistance. These systems are increasingly used in real-world scenarios. Compared to traditional automated equipment, this translates into enhanced operational flexibility and intelligence. Accordingly, their navigation and obstacle-avoidance performance not only underpins the efficiency of their operations but also serves as a key indicator of their autonomy and intelligence levels.

At present, dynamic obstacle-avoidance methods based on the Robot Operating System (ROS), such as the Dynamic Window Approach (DWA), have become widely adopted technical solutions for enabling efficient navigation in mobile robots. These approaches have demonstrated promising results in experimental settings and offer substantial value for practical engineering applications [1]. However, in real-world deployment scenarios, mobile robots often face challenges, such as incomplete perception data, accumulated mapping errors, delayed path re-planning, and unexpected obstacles. Collectively, these factors reduce the adaptability of robotic systems in dynamic environments and compromise the overall robustness of their navigation capabilities [2]. In response, researchers and engineers are working to improve obstacle-avoidance mechanisms for better efficiency, stability, and real-time responsiveness. Multimodal strategies that combine deep Reinforcement Learning, visual perception, obstacle prediction, and trajectory optimisation have emerged as prominent research directions [3,4].

Among the various robot operating systems, ROS has been widely adopted for the development and research of mobile robot navigation systems, owing to its modular architecture, hardware–software decoupling, and comprehensive development toolchain. Its core navigation framework, commonly referred to as the ROS navigation stack, integrates key functional modules, including Simultaneous Localisation And Mapping (SLAM), localisation (e.g., AMCL), global and local path planning, obstacle-avoidance control, and recovery behaviours. This architecture provides a complete closed-loop system that supports autonomous navigation in mobile robots [5].

With the release of ROS 2, significant advancements have been made in communication architecture, real-time performance, and cross-platform deployment capabilities [6]. The Nav2 framework, based on a Behaviour Tree scheduling mechanism, adopts a plugin-based architecture that facilitates flexible integration and switching among various global planners (such as Hybrid A*, Smac Planner, and State Lattice) and local controllers (such as DWB, TEB, and MPPI). This design greatly enhances the system’s scalability and adaptability across a wide range of navigation scenarios [7,8].

The Dynamic Window Approach (DWA) is widely employed in low-to-medium-speed autonomous navigation tasks, due to its low computational cost and strong real-time performance. As demonstrated in the study by Anh-Tu Nguyen et al. [1], the ROS-based implementation of DWA, when integrated with 2D grid costmaps and LiDAR sensors, can effectively avoid both static and moderately dynamic obstacles. The experimental results have confirmed that this method offers reliable real-time responsiveness and low computational complexity, making it well suited for autonomous navigation in complex environments at relatively low speeds.

However, traditional path-planning algorithms, such as DWA, often experience delayed responsiveness and rigid trajectory behaviour when confronted with high-speed dynamic obstacles. To overcome these limitations, researchers have explored the integration of deep learning techniques with conventional planning strategies to improve generalisation capability and adaptability in dynamic scenarios. For example, Jin et al. proposed a deep Reinforcement Learning algorithm, LB-DDQN, which incorporates B-spline curve optimisation to enhance path smoothness and substantially increase responsiveness to fast-moving obstacles [2].

In addition, Sousa et al. integrated YOLOv3-based visual detection with a fuzzy control strategy to support real-time obstacle avoidance in collaborative human–robot environments. This approach improved safety and robustness under dynamic human interference [9]. In more complex dynamic environments, Chen et al. proposed a multi-target tracking and velocity-estimation mechanism based on extended Kalman filtering and LiDAR point clouds. Integrated with the Timed Elastic Band (TEB) planner, this method optimised the system’s trajectory-adjustment strategy and improved obstacle-avoidance performance, achieving a 21.05% success rate increase [8]. Similarly, Gao et al. developed a hybrid planning strategy combining an improved A* algorithm with DWA, incorporating obstacle inflation and global distance evaluation to enhance rationality and robustness in dense environments such as photovoltaic power stations [10]. Furthermore, Chen C.S. et al. introduced a velocity obstacle layer within the ROS costmap framework, enabling real-time estimation of obstacle velocities and applying truncated area optimisation to anticipate interference. This significantly improved the responsiveness and flexibility of local planners such as DWA [3].

The development of navigation and obstacle-avoidance systems is steadily progressing from traditional reactive methods towards predictive and optimisation-based strategies [7]. Modern system design increasingly emphasises the integration of multi-source perception, human behaviour modelling, and adaptive path control, aiming to achieve highly robust and intelligent navigation in complex, dynamic environments.

This study aims to provide a clear summary of recent research on ROS-based navigation stacks and obstacle avoidance. It focuses on system architecture, core algorithms, real-world challenges, and the integration of advanced technologies. It also provides technical insights and references for both researchers and developers, facilitating the practical deployment and continuous evolution of ROS-based navigation systems across diverse scenarios and platforms.

The remainder of this study is structured as follows. Section 2 introduces the fundamental architecture of the ROS navigation stack and outlines its evolution from ROS 1 to ROS 2, with emphasis on modular design, communication frameworks, and scheduling mechanisms. Section 3 presents a comprehensive analysis and comparison of representative obstacle-avoidance algorithms implemented in ROS, including heuristic methods, optimisation-based techniques, and sampling-based approaches. Section 4 discusses the main technical challenges encountered in practical applications, such as limitations in perception accuracy, reduced robustness in dynamic environments, difficulties in multi-robot coordination, and challenges in cross-platform deployment. Section 5 examines emerging research directions, including the integration of Artificial Intelligence into navigation, collaboration between cloud and edge computing, socially aware navigation behaviours, and the industrial deployment of ROS 2 systems. Finally, Section 6 concludes the paper and suggests potential directions for future research.

This study distinguishes itself in three main aspects. First, it systematically analyses the evolution from ROS 1 to ROS 2 navigation stacks, focusing on communication architecture, Behaviour Tree scheduling, and plugin-based modularity. Second, it goes beyond traditional planners (DWA, TEB, MPPI) by integrating and comparing data-driven methods such as Reinforcement Learning and vision-based navigation. Third, it comprehensively discusses deployment challenges across multi-robot systems, human–robot interaction, and resource-limited platforms, while highlighting emerging trends like semantic perception and social behaviour modelling. These perspectives offer a more integrated and forward-looking reference than existing ROS navigation reviews.

## 2. ROS System Architecture

As noted above, autonomous navigation capability is a fundamental prerequisite for mobile robots to perform tasks efficiently and adapt to diverse environments in applications such as industrial automation, warehouse logistics, and service robotics. As an open-source middleware framework, ROS provides a comprehensive navigation stack that integrates environmental perception, map construction, path planning, and motion control functionalities. This stack has been widely adopted in both academic research and industrial deployment contexts, supporting a wide range of platforms and robotic systems [11,12,13].

With increasing environmental dynamics, the emergence of multi-robot systems, and rising demands for real-time performance, the traditional ROS 1 navigation architecture has revealed several limitations, in terms of communication mechanisms, modular extensibility, and task scheduling. This section examines the architectural evolution of the ROS navigation stack under both the ROS 1 and ROS 2 frameworks, highlighting key technical modules and recent advancements. The discussion provides a technical foundation for the subsequent analysis of obstacle-avoidance mechanisms and system-level integration strategies.

### 2.1. Architecture of the ROS Navigation Stack

**Figure 1:**
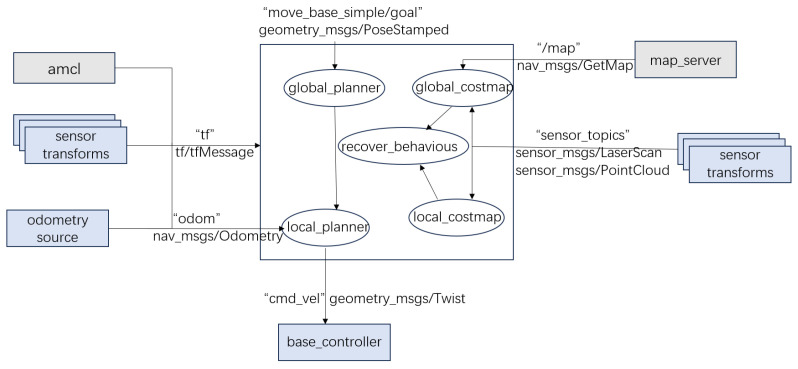
Module communication architecture of the ROS navigation stack.

The ROS navigation stack is typically composed of five core functional layers: perception, state estimation, map modelling, path planning, and control execution. These modules are interconnected via ROS’s publish–subscribe communication mechanism, which enables modular decoupling, plugin replacement, and runtime parameter tuning. This design offers excellent system extensibility and adaptability across a wide range of application scenarios [14,15]. A typical communication structure among these modules is illustrated in Figure 1. The principal components of each functional layer are as follows:

The perception layer is responsible for multi-sensor data acquisition and preprocessing. It typically integrates sensors such as 2D LiDAR, RGB-D cameras, and Inertial Measurement Units (IMUs) to construct environmental point clouds and provide obstacle information as input to the navigation system. Several studies have proposed methods that fuse depth images from RGB-D sensors with LiDAR projection data, significantly improving mapping accuracy and enhancing the robustness of obstacle detection [15,16].

The state estimation layer estimates the robot’s pose by fusing data from Inertial Measurement Units (IMUs), odometry, and visual or LiDAR-based sensors. Commonly used approaches include Adaptive Monte Carlo Localisation (AMCL), the Extended Kalman Filter (EKF), and graph optimisation-based methods such as RTAB-MAP. RTAB-MAP has been widely applied for accurate localisation in dynamic or large-scale environments, and it is often integrated with graph optimisation frameworks, such as GTSAM and TORO, to enforce error constraints and improve estimation consistency [14,15,16].

The map modelling layer provides the robot with spatial awareness of its environment. It supports both loading pre-built static maps and constructing dynamic maps using SLAM techniques such as slam_toolbox and RTAB-MAP. Numerous studies have demonstrated that combining RGB-D data with LiDAR measurements facilitates the construction of high-precision 2D or 3D map models, which effectively supports subsequent path-planning tasks.

The path-planning layer typically comprises two components: global path planning (e.g., A*, Dijkstra) and local obstacle avoidance (e.g., DWA, TEB). These algorithms dynamically generate optimal trajectories based on the current map and obstacle information. They also support real-time re-planning capabilities, enabling the robot to perform effective dynamic obstacle avoidance in complex environments [14].

The control execution layer is responsible for translating planned trajectories into concrete motion control commands. It typically publishes linear and angular velocity instructions via the standard ROS interface cmd_vel, forming a closed-loop with the robot’s base controller. Related system designs have shown that controller plugins can be adapted to various chassis models and support dynamic parameter tuning to meet the requirements of different task environments [15].

**Figure 2:**
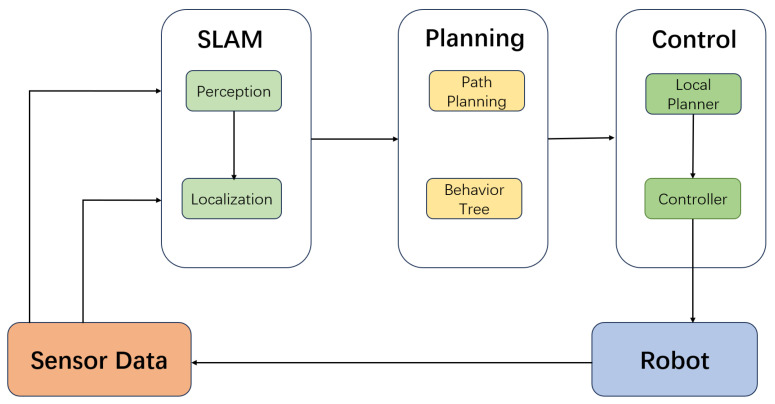
High-level functional architecture and data flow of the ROS navigation stack.

Figure 2 illustrates the high-level functional architecture of the ROS navigation stack, outlining the interactions among the perception, localization, planning, and control modules.

### 2.2. Architectural Differences and Optimizations Between ROS 1 and ROS 2

To address the limitations of ROS 1—such as communication bottlenecks caused by its centralised architecture, strong coupling between modules, and the complexity of debugging—ROS 2 introduces several key architectural optimisations. One of the most significant enhancements is the transition from the original TCPROS protocol to a Data Distribution Service (DDS)-based distributed communication architecture. This enables low-latency, high-reliability messaging with configurable Quality of Service (QoS) policies, making ROS 2 well suited for real-time and safety-critical industrial applications [17].

ROS 2 also introduces a lifecycle node management mechanism, which allows dynamic control over the operational states of modules (e.g., activation, suspension, and destruction). This significantly improves the system’s fault tolerance and flexibility. Within the navigation stack, core functional modules, such as planners, controllers, and recovery behaviours, are implemented using a plugin-based architecture, thereby enhancing the system’s modularity, re-usability, and extensibility [18].

In addition, ROS 2 employs Behaviour Trees (BTs) for task scheduling and behaviour control, supporting conditional branching, priority-based execution, and multi-level fault recovery. This design offers strong robustness and flexibility in dynamic environments [13].

To further compare the differences between ROS 1 and ROS 2, in terms of system architecture, communication mechanisms, and functional extensibility, this study summarises the key distinctions across several critical dimensions, as presented in Table 1.

As shown in Table 1, ROS 2 enhances system decoupling and real-time performance, while preserving compatibility with ROS 1 [6,13]. These improvements provide a more flexible and robust architectural foundation for mobile robotic systems.

### 2.3. Behaviour Tree–Driven Navigation Control Workflow

Behaviour Trees (BTs) serve as the core scheduling structure in the ROS 2 navigation system (Nav2), replacing traditional Finite State Machines (FSMs). BTs offer a clear hierarchical structure, strong extensibility, and robust task-recovery capabilities. Navigation tasks are defined in XML format, in which core behaviour nodes, such as NavigateToPose, FollowPath, and BackUp, interact with key components, including the Planner Server, Controller Server, and Recovery Server. This design enables a unified workflow that seamlessly integrates path planning, trajectory tracking, and failure handling.

**Figure 3:**
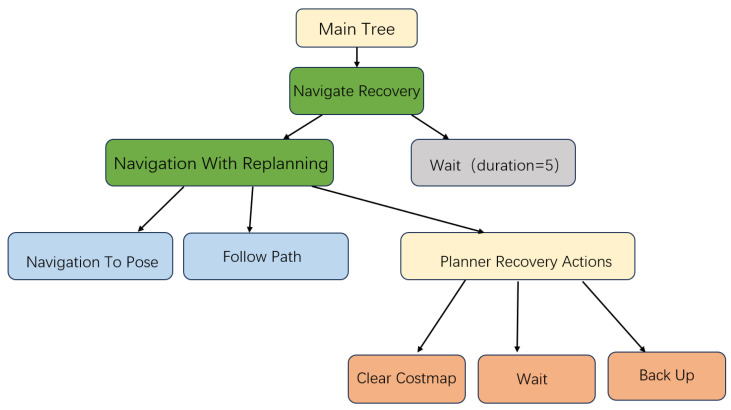
Task scheduling and recovery in Nav2 via Behaviour Trees.

The overall workflow of task scheduling and recovery in Nav2 is illustrated in Figure 3.

Compared to Finite State Machines (FSMs), Behaviour Trees (BTs) offer several distinct advantages:

- High re-usability of nodes: behaviour nodes are re-usable across different tasks, eliminating the need to redesign the control model.
- Support for asynchronous event handling and hierarchical fault recovery: BTs are capable of monitoring dynamic environmental changes in parallel and executing recovery actions in a hierarchical manner when failures occur.
- Ease of visual debugging: the tree-based structure is intuitive and transparent, and supported visualisation tools enable real-time monitoring of node states, significantly simplifying debugging and maintenance.

Recent studies have begun to explore the integration of BTs with Reinforcement Learning and task graphs to further enhance the intelligence and adaptability of behaviour scheduling [13,18].

### 2.4. Core Technology Module Analysis

#### 2.4.1. State Estimation and Localisation

Mainstream localisation methods in ROS navigation systems include the following:

- HAMCL (Adaptive Monte Carlo Localisation): suitable for known-map scenarios, this method employs particle filtering to fuse laser scan data and odometry for accurate pose estimation.
- SLAM algorithms: solutions such as Gmapping, Cartographer, and slam_toolbox support simultaneous localisation and mapping, making them well suited for operation in unknown environments.

To enhance localisation robustness in dynamic environments, Abaza et al. proposed an AI-driven dynamic covariance modelling approach that integrates machine learning into the ROS robot_localisation package. This method adaptively tunes the covariance parameters of the Extended Kalman Filter (EKF), effectively suppressing pose drift errors [19].

#### 2.4.2. Path Planning and Tracking Control

Global planning: in static grid maps, A* and Dijkstra’s algorithm are widely used for global path planning. Dijkstra’s algorithm guarantees an optimal path by exhaustively exploring all nodes, making it suitable for small-scale environments [20]. In contrast, A* combines path cost with a heuristic function to significantly improve search efficiency [21]. Ou, Y. et al. proposed an improved version of the A* algorithm for 2D static maps, incorporating path smoothing and turning cost models to enhance both path quality and computational performance [22]. These studies confirm the effectiveness of both algorithms in generating the shortest paths under static environmental conditions.

Local planning: DWA and TEB are commonly employed for real-time obstacle avoidance and trajectory optimisation. Among them, the Timed Elastic Band (TEB) algorithm is particularly well suited for environments with dynamic obstacles, as it models the robot’s path as an elastic band subject to both spatial and temporal constraints [23]. TEB dynamically adjusts trajectory nodes to accommodate changes in obstacle distribution. In recent years, researchers have further enhanced TEB by introducing variable splitting techniques [24], social navigation mechanisms [25], and Reinforcement Learning strategies [26], significantly improving its robustness and adaptability in dynamic environments. These developments underscore TEB’s strong potential for enabling efficient and safe navigation in complex real-world scenarios.

Advanced algorithms: in the field of path planning for dynamic environments, the Strategy-based Dynamic Object Velocity Space (S-DOVS) model introduces obstacle velocity vectors to construct a velocity space representation that integrates both spatial and temporal characteristics. This approach significantly improves the rationality of local path generation and the success rate of obstacle avoidance. Studies have shown that S-DOVS can perceive the motion trajectories of dynamic obstacles in real time and, by incorporating the kinematic and dynamic constraints of mobile robots, generate safe and feasible velocity commands. It is particularly well suited for local path-planning tasks in highly dynamic and unstructured environments. Building on this foundation, researchers have further explored the integration of Reinforcement Learning into the DOVS framework to enhance system adaptability in complex environments. The resulting method, RL-DOVS, incorporates a Q-learning strategy into the S-DOVS model, enabling the system to autonomously learn optimal obstacle-avoidance behaviours within the dynamic velocity space. The experimental results have demonstrated that RL-DOVS exhibits stronger behavioural generalisation and higher avoidance efficiency in high-density dynamic obstacle scenarios, marking a significant step towards intelligent and self-learning navigation strategies [27].

#### 2.4.3. Obstacle-Avoidance Mechanisms and Costmaps

In the ROS navigation system, the costmap_2d module is commonly used to construct both global and local costmaps. Its core structure typically consists of three layers:

- The static layer is responsible for loading predefined static obstacle information;
- The obstacle layer updates the environmental model in real time based on perception data from LiDAR or depth cameras;
- The inflation layer expands the boundaries of detected obstacles according to safety distance requirements, ensuring the feasibility and safety of path planning.

To address navigation stability issues in dynamic and complex environments, Reference [13] proposed a Behaviour Tree–based control architecture that encapsulates obstacle detection and path recovery as functional nodes, thereby enabling flexible task switching and enhanced fault tolerance during navigation.

Furthermore, to meet the social behaviour requirements of human–robot collaborative environments, related studies have introduced the Proxemic Layer mechanism, which models human social space boundaries within the costmap. This approach enhances the robot’s behavioural adaptability and compliance with social norms when operating in human-centred environments [13,23].

#### 2.4.4. Controller Execution and Task Interfaces

The controller generates velocity commands based on the local trajectory and drives the robot’s movement through an execution mechanism. The controller plugins within the Nav2 framework support various types of mobile bases, such as differential drive, omnidirectional Mecanum wheels, and Ackermann steering, and they allow switching between different control models, including classical PID control and Nonlinear Model Predictive Control (NMPC) [13].

As a recent research focus, NMPC offers both high trajectory-tracking accuracy and strong obstacle-avoidance capabilities, making it well suited for omnidirectional platforms and complex, dynamic environments.

This section systematically reviews the core modular structure and technological evolution of the ROS navigation stack, providing a comprehensive analysis of the implementation mechanisms and performance characteristics of its five major functional layers—perception, localisation, mapping, path planning, and control execution—as applied under both the ROS 1 and ROS 2 frameworks. In particular, the Behaviour Tree-based scheduling mechanism introduced in ROS 2 enables a high degree of decoupling and intelligent control for navigation tasks, significantly enhancing system scalability, fault tolerance, and behavioural flexibility. Furthermore, this section explores state estimation methods, global and local path-planning algorithms, costmap construction strategies, and controller execution models, highlighting the ROS navigation system’s integrated capabilities in multi-source data fusion, real-time obstacle avoidance, and multi-platform adaptability.

In emerging areas, such as AI-assisted state estimation, deep Reinforcement Learning-driven path optimisation, and the integration of decision-making and perception layers, the ROS navigation system has achieved promising results, demonstrating increasing robustness and adaptability in dynamic, complex, and human–robot collaborative environments. Looking ahead, with the advancement of edge computing, the maturation of multi-robot collaboration mechanisms, and the widespread adoption of high-level intelligent technologies such as semantic perception and contextual reasoning, the ROS navigation stack is expected to continue evolving. It is anticipated to support large-scale heterogeneous system deployments, automatic task adaptation, and improved understanding of human proxemics, thereby ushering in a new era of more intelligent, efficient, and collaborative autonomous navigation for mobile robots.

## 3. Obstacle-Avoidance Algorithms

### 3.1. Definition of Obstacle-Avoidance Tasks and Key Technical Challenges

Obstacle avoidance is typically defined as the process by which a mobile robot, while navigating towards a target position, relies on real-time environmental perception to detect and avoid both static and dynamic obstacles. The robot dynamically generates safe and feasible trajectories that comply with its kinematic and dynamic constraints [28]. Within the ROS system architecture, this task is primarily carried out by the local path planner, which adjusts the trajectory and generates control commands based on sensor inputs such as LiDAR and depth cameras. This enables the robot to autonomously avoid obstacles within its surrounding environment.

The main technical challenges currently faced in obstacle avoidance can be summarised as follows:

- Incomplete perception and inconsistent sensor fusion: although integrating multiple sensors (such as ultrasonic sensors, LiDAR, and cameras) can enhance the comprehensiveness and robustness of environmental modelling, differences in perception range and measurement accuracy among sensors often lead to incomplete coverage or fusion bias during real-world deployment scenarios [28].
- Uncertainty introduced by dynamic obstacles: in dynamic environments, the behaviour of obstacles is often unpredictable, requiring frequent path re-planning to adapt to changes in both targets and obstacles. This introduces significant uncertainty and places high demands on the real-time responsiveness of the path-planning system [29].
- Trade-off between real-time performance and optimality: under embedded deployment conditions, it is necessary to balance the trade-off between algorithmic solving speed and the quality of locally optimal trajectories [30].
- System robustness and limited transferability: perception errors, map inconsistencies, and unexpected obstacles can result in the failure of the path-planning strategy. Therefore, it is essential to improve the system’s adaptability to perception instability and its transferability to new environments [31].

### 3.2. Typical Paradigms and Comparative Analysis of Obstacle-Avoidance Algorithms in ROS

In the ROS navigation system, obstacle-avoidance mechanisms are primarily based on local path planning. Classical approaches can be categorised into three main types: heuristic methods (e.g., Dynamic Window Approach, DWA), optimisation-based methods (e.g., Timed Elastic Band, TEB), and sampling-based predictive methods (e.g., Model Predictive Path Integral Control, MPPI). Each of these methods exhibits distinct characteristics, in terms of planning efficiency, environmental adaptability, and control accuracy, constituting the core evolutionary trajectory of ROS-based obstacle-avoidance systems.

#### 3.2.1. Dynamic Window Approach (DWA)

As introduced in Section 2.4, the Dynamic Window Approach (DWA) generates feasible trajectories in real time by sampling the velocity space and evaluating each option based on cost functions involving heading alignment, obstacle proximity, and velocity efficiency [32].

**Figure 4:**
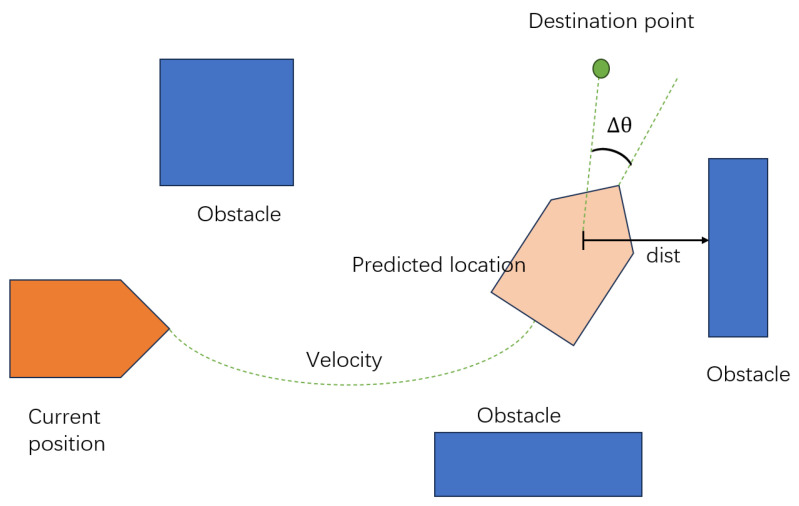
Schematic illustration of trajectory prediction and cost evaluation in the Dynamic Window Approach algorithm.

As illustrated in Figure 4, the algorithm predicts the robot’s future positions based on its current velocity, and it then computes the heading deviation (Δθ) and the minimum distance to nearby obstacles (dist) to guide trajectory selection and control command generation.

DWA has been integrated as the default local planner in the ROS move_base module, due to its simplicity and computational efficiency. However, it offers limited adaptability in highly dynamic environments. To address this, recent studies have explored trajectory prediction models and obstacle behaviour modelling [29,33], as well as Reinforcement Learning strategies for adaptive cost parameter tuning [31,34].

#### 3.2.2. Timed Elastic Band (TEB)

The TEB algorithm, previously discussed in Section 2.4, formulates the local trajectory as a time-parameterised elastic band, optimising path smoothness, temporal distribution, and obstacle constraints under a nonlinear optimisation framework [10].

**Figure 5:**
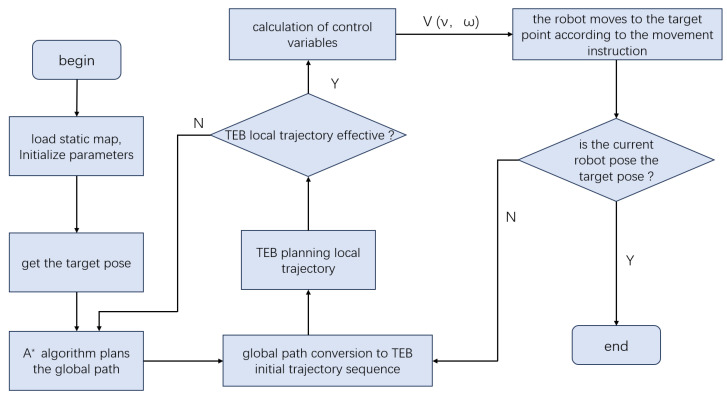
Workflow of the Timed Elastic Band (TEB) algorithm for trajectory tracking.

As shown in Figure 5, TEB transforms the global path into an initial local trajectory, iteratively refines it based on real-time perception data, and outputs motion commands compliant with kinematic and dynamic constraints.

Although TEB performs reliably in static or structured environments, its effectiveness depends heavily on the quality of the global planner’s guidance and the real-time accuracy of the sensor inputs. In dynamic and complex scenarios, TEB may exhibit delayed path responsiveness and a reliance on reverse manoeuvres, which can adversely affect continuity and navigation efficiency. Therefore, in practical applications TEB is often combined with global path-planning algorithms, such as A* or Dijkstra, in order to enhance the overall robustness and adaptability of the navigation system [35].

#### 3.2.3. Model Predictive Path Integral (MPPI)

Model Predictive Path Integral (MPPI) control integrates path integral optimal control theory with the Model Predictive Control (MPC) framework. During each control cycle, MPPI samples a large number of control sequences and evaluates their associated costs, selecting the optimal control action through a weighted averaging process [36]. MPPI demonstrates strong capabilities in modelling nonlinear dynamics and can naturally handle high-dimensional state constraints, making it particularly suitable for complex navigation tasks characterised by high dynamics and significant uncertainty.

However, due to its reliance on large-scale sampling and online optimisation, MPPI faces certain limitations, in terms of real-time performance and computational overhead. To address these challenges, recent research has proposed various improvements aimed at enhancing the practicality and robustness of MPPI:

- GP-MPPI introduces Gaussian Process modelling to capture dynamic environmental state transitions, thereby enhancing the system’s responsiveness to moving obstacles and improving overall navigation robustness [37].
- DRPA-MPPI incorporates a Dynamic Repulsive Potential Approach (DRPA) to strengthen local obstacle-perception and -avoidance capabilities, particularly in unknown or unstructured environments [38].
- Hybrid A-MPPI* integrates globally guided paths generated by the Hybrid A* algorithm, thereby improving MPPI’s consistency with global navigation goals and its performance in complex map scenarios [39].

### 3.3. Advances in Data-Driven Obstacle-Avoidance Methods

In addition to the aforementioned classical planning methods, data-driven approaches, such as Reinforcement Learning and deep learning, have demonstrated increasingly promising performance in obstacle-avoidance tasks in recent years, and they have emerged as a significant extension of obstacle-avoidance techniques within the ROS ecosystem. Implementation resources and links for selected frameworks are provided in Appendix A.

#### 3.3.1. Reinforcement Learning

Reinforcement Learning (RL) enables robots to autonomously learn obstacle-avoidance strategies through trial-and-error interactions and policy iteration, thereby establishing a state–action mapping. Recent studies have applied deep Reinforcement Learning methods, such as Deep Q-Networks (DQN) and Proximal Policy Optimisation (PPO), within ROS-based simulation environments including Gazebo. These approaches are often integrated with traditional planners (e.g., DWA) to enhance system flexibility and adaptability [31,34].

#### 3.3.2. Vision-Based Deep Learning Methods

Visual navigation relies on Convolutional Neural Networks (CNNs) or Transformer-based models to extract semantic information from images, enabling the identification of traversable areas or the direct regression of control commands. Open-source frameworks such as Arena-Rosnav have successfully integrated deep learning-based policies into ROS systems, demonstrating strong generalisability and scalability across diverse scenarios [35,40].

To address the sensitivity of purely vision-based approaches to lighting conditions and occlusions, some studies have incorporated data from additional sensors, such as LiDAR and millimetre-wave radar. This multimodal fusion strategy significantly enhances the reliability of obstacle detection and avoidance in complex environments [29].

These three classical paradigms—namely DWA, TEB, and MPPI—represent some of the most widely adopted local planning strategies within the ROS navigation framework. Each method involves distinct trade-offs, in terms of computational cost, planning robustness, and environmental suitability. To capture a broader range of approaches, recent advancements, such as Reinforcement Learning and vision-based planning, have also been considered, reflecting growing trends in data-driven and perception-enhanced navigation. A qualitative comparison of these classical and learning-based methods is summarised in Table 2, while Table 3 provides a corresponding quantitative evaluation based on latency, success rate, environmental applicability, and computational performance.

## 4. Problems and Challenges

As ROS-based navigation systems become more widely deployed, researchers have raised concerns about their reliability in unstructured environments [41,42]. This section systematically analyses the current technical challenges faced by the ROS navigation stack and its obstacle-avoidance mechanisms from five key perspectives: perception and localisation errors, navigation robustness, resource constraints, multi-robot coordination, and cross-platform deployment.

### 4.1. Error Analysis of Perception and Localisation Technologies

Mobile robot navigation heavily relies on environmental perception and self-localisation. However, during real-world operation, perception errors and localisation drift often lead to path deviation and obstacle misdetection. Studies have shown that commonly used localisation methods, such as AMCL, perform unreliably in environments with sparse features or complex lighting conditions, frequently resulting in particle degeneration and sudden pose jumps [19,41]. Meanwhile, although Karto SLAM achieves high accuracy in small-scale environments, it tends to suffer from mismatches in large-scale scenes, potentially causing failures in global path reconstruction, with maximum relative errors reaching up to 4.69% [42].

In addition, some studies highlight that dynamic environments pose significant challenges to the stability of SLAM systems. For instance, pedestrian movement or object relocation can lead to map mismatches [43]. To improve perception reliability in such scenarios, Reference [44] proposed a multimodal fusion approach that combines YOLOv7-based object detection with LiDAR data, significantly enhancing obstacle recognition accuracy and map consistency. Furthermore, Reference [45] demonstrated that integrating deep learning with radar-based mapping improves the system’s tolerance to local occlusions.

To enhance 3D environment mapping capabilities, researchers have proposed using RGB-D cameras to acquire high-resolution point clouds and construct costmaps [7,46]. Such methods effectively detect low-profile obstacles and suspended objects, thereby improving the expressiveness of ROS costmaps in representing complex terrain.

### 4.2. Navigation Robustness in Dynamic and Complex Environments

In dynamic and unstructured environments, the robustness of robotic navigation systems is challenged by factors such as moving obstacles, map changes, and sensor delays. Traditional algorithms (e.g., DWA) maintain relatively stable paths in low-density obstacle environments; however, they often suffer from path oscillations and local minima issues under dense dynamic interference.

As noted in [47], although Reinforcement Learning-based obstacle-avoidance methods can be trained to acquire complex strategies, their generalisation capability remains limited, potentially leading to navigation failures when encountering previously unseen obstacle behaviours.

To address the aforementioned issues, researchers have proposed a hybrid navigation strategy that combines YOLOv7 with Rapidly-exploring Random Trees (RRTs), known as NAV-YOLO-RRT. This approach enables real-time tracking of dynamic targets and adaptive path reconstruction, significantly improving obstacle-avoidance success rates [44]. In another study focused on collaborative warehouse environments, a “virtual safety zone” model was introduced. This model utilises YOLOv3 to detect human positions and employs a fuzzy controller to dynamically adjust the robot’s speed, thereby enhancing safety in human–robot coexistence scenarios [7].

In addition, studies [43,48] have highlighted that when robots navigate in densely populated areas, it is essential to respond proactively to human behaviours. Traditional purely reactive obstacle-avoidance strategies often struggle to meet the dual demands of rapid responsiveness and optimal path planning. Therefore, it is necessary to integrate visual recognition and trajectory prediction methods to enhance the system’s adaptability in complex crowd scenarios.

### 4.3. Technical Adaptation Challenges on Resource-Constrained Platforms

Mobile robots are often deployed on low-power embedded platforms, such as TurtleBot3 or Jetson Nano, where limited computational resources make it difficult to execute computation-intensive tasks, such as deep learning, global path optimisation, and graph-based SLAM, in real time. Reference [44] proposed the use of a lightweight YOLOv7-Tiny model as the primary perception backbone, significantly reducing computational overhead while maintaining acceptable detection accuracy. Reference [7] demonstrated the integration of YOLOv3-Tiny with a fuzzy controller on the KUKA Youbot platform, achieving real-time performance with smooth obstacle avoidance in Gazebo simulations.

Reference [46] further demonstrated that combining RGB-D cameras with lightweight Convolutional Neural Networks (CNNs) can offload the sensing burden from LiDAR, making such systems more feasible for deployment on embedded platforms with limited computational resources. Reference [49] introduced the FogROS2 framework, which enables dynamic offloading of compute-intensive tasks, such as image recognition and path planning, to edge or cloud infrastructure. This architecture significantly enhances system execution efficiency while maintaining real-time capabilities, achieving up to a 45× speed-up in SLAM reconstruction.

However, such cloud-dependent systems also introduce potential challenges related to communication delays and network reliability, particularly in time-critical scenarios such as emergency obstacle avoidance. Reference [45] emphasised that a balanced partitioning of local and cloud-based modules is crucial for achieving efficient and stable collaborative navigation in multi-robot systems.

### 4.4. Multi-Robot Collaborative Navigation Challenges

In multi-robot systems, achieving collaborative navigation requires addressing challenges such as path conflicts, task overlap, and the maintenance of map consistency. Reference [48] provides a comprehensive review of multi-robot map merging, local path reconstruction mechanisms, and fault tolerance under communication interruptions. It also notes that heterogeneous map fusion and behaviour coordination strategies remain in an exploratory phase and require further investigation.

Reference [44] proposed an improved obstacle-avoidance framework that combines YOLO with RRT, specifically designed for deployment in multi-robot systems. By sharing visual recognition results and dynamically adjusting local trajectories, the model effectively reduces path conflicts and resource competition, thereby enhancing overall navigation efficiency and cooperative robustness. However, as highlighted in previous studies [43], communication interruptions and robot disconnections may still compromise system-level scheduling. To address these issues, decentralised coordination strategies and task reallocation mechanisms are needed to improve system resilience.

Additionally, Reference [50] explores architectural solutions for maintaining consistency and security in multi-platform ROS 2 deployments, recommending Kubernetes-based node isolation and WireGuard-encrypted communication to ensure reliable task execution and data integrity in distributed multi-robot systems.

### 4.5. Technical Bottlenecks in Cross-Platform Deployment

When migrating ROS systems from development platforms to industrial-grade or embedded environments, developers often encounter several challenges, including dependency conflicts, incompatibilities in hardware abstraction layers, and the need to restructure communication interfaces. Reference [50] presents a systematic analysis of Nav2 deployments on both x86 and ARM architectures, indicating that although containerisation improves portability, real-time critical tasks still require enhancements, such as node prioritisation mechanisms and real-time kernel optimisations, in order to meet scheduling requirements.

The FogROS2 architecture offers a promising approach for cross-platform deployment. As described in Reference [49], the integration of Kubernetes with WireGuard enables secure and modular deployment of heterogeneous ROS containers, supporting inter-node communication across resource-constrained platforms. Through remote configuration synchronisation and ROS parameter re-mapping, this method ensures functional consistency and compatibility across diverse hardware systems.

Furthermore, Reference [45] highlights additional barriers encountered in highly customised hardware systems, such as industrial AGVs and collaborative robotic arms, where proprietary sensor drivers or specialised hardware interfaces are not always supported. In such cases, the development of ROS-to-hardware middleware interfaces becomes necessary to bridge compatibility gaps, although this often comes at the cost of increased system integration complexity.

Despite recent advancements, ROS-based navigation systems continue to face notable challenges in perception accuracy, navigation robustness, cross-platform deployment, and multi-robot coordination. In dynamic and unstructured environments, issues such as response latency, limited path reconfiguration, and insufficient perception fault tolerance remain prominent. Future research should focus on enhancing sensor fusion reliability, developing adaptive navigation algorithms, and implementing decentralized coordination strategies. Moreover, addressing computational limitations on embedded platforms is essential to enable lightweight and modular system architectures suitable for real-world deployment.

## 5. Discussion and Emerging Trends

With the widespread application of robotic technologies across diverse domains, including agriculture, industry, urban environments, and service sectors, ROS-based navigation stacks and obstacle avoidance mechanisms are undergoing a critical transition from traditional modular approaches to more intelligent, collaborative, and industrial-grade architectures. Based on a comprehensive review of recent domestic and international research, this section systematically outlines the current evolution and future trends in navigation technologies, focusing on four key directions: the integration of Artificial Intelligence, cloud–edge collaborative frameworks, human–robot interaction, and industrial deployment on the ROS 2 platform.

### 5.1. Deep Integration of Navigation Technologies and Artificial Intelligence

Conventional path-planning approaches in the ROS navigation stack, such as the Dynamic Window Approach and Timed Elastic Band, primarily rely on manually designed heuristics or optimisation-based models. While these methods demonstrate stable performance in static environments, they face significant limitations in responsiveness and generalisation when deployed in unstructured or dynamic settings.

In recent years, Artificial Intelligence technologies, including deep learning and Reinforcement Learning, have been progressively integrated into ROS-based navigation systems, enabling end-to-end perception and decision-making. This shift represents a new driving force in the evolution of intelligent navigation.

As a representative example, Zhao et al. [51] proposed an end-to-end deep Reinforcement Learning framework for autonomous UAV landing within an ROS2-based system. The approach utilises monocular visual input and the DDPG algorithm to directly generate velocity control commands from onboard camera data. By bypassing traditional modules, such as mapping and planning, the system significantly simplifies the navigation pipeline and improves real-time responsiveness. The simulation results in Gazebo have demonstrated robust landing performance, even under initial yaw deviations or lateral offsets, highlighting the feasibility of integrating deep RL into practical ROS navigation scenarios.

The integration of Artificial Intelligence into navigation systems has significantly enhanced robotic perception and decision-making capabilities, marking a paradigm shift from rule-based to data-driven approaches [52]. However, this evolution also introduces several challenges, including high computational demands, limited generalisation across diverse environments, and the complexity of model training.

Future research could focus on the development of lightweight models, cross-domain generalisation strategies, and the incorporation of human behaviour learning mechanisms, with the aim of improving the robustness and adaptability of autonomous navigation in real-world scenarios [53,54].

### 5.2. Cloud–Edge Collaborative Navigation Architecture

Jin et al. [55] developed an ROS 2-based navigation framework tailored for horticultural scenarios. In this system, LiDAR data collected by field robots is transmitted to the cloud, where high-precision semantic maps are constructed to support global path planning. The computed paths are then sent back to the robot, where local motion control and obstacle avoidance are executed on onboard edge computing units. The system was deployed and tested in semi-structured agricultural fields, achieving approximately 36 ms average tracking latency and a 100% path success rate. This case demonstrates the practical feasibility of cloud–edge collaborative navigation under real-world deployment conditions.

Seisa et al. [56] proposed an edge-based architecture for offloading Model Predictive Control (MPC) tasks from UAVs to edge servers, significantly reducing on-board computational load and latency. Their work demonstrates the feasibility of integrating edge computing into real-time autonomous navigation, offering a viable solution framework for cloud–edge collaborative navigation systems under resource-constrained conditions.

Singh et al. [57] provided a comprehensive review of deep Reinforcement Learning algorithms in mobile robot navigation, highlighting their superior adaptability in dynamic and uncertain environments. Within a cloud–edge collaborative architecture, integrating such learning-based strategies at the edge layer enables real-time policy updates and local adaptation, thereby enhancing the robustness of navigation systems.

The native support for distributed architecture, Quality-of-Service (QoS) policies, and multi-node coordination in ROS 2 provides essential infrastructure for building cloud–edge collaborative navigation systems. Future research will focus on key challenges, such as task coordination across heterogeneous nodes, autonomous fault tolerance, and predictive task scheduling, with the aim of further enhancing the intelligence, resilience, and scalability of navigation systems within cloud–edge–device architectures.

### 5.3. Navigation Technologies for Human–Robot Interaction and Collaboration

In densely populated environments, such as hospitals and shopping centres, robots with basic obstacle-avoidance capabilities often exhibit rigid behaviour that may cause discomfort or even conflict with pedestrians. As a result, Socially Aware Navigation (SAN) is emerging as a key research focus within ROS-based navigation systems.

The HuNavSim platform [52], developed based on ROS 2 and Gazebo, enables simulation of human behaviours such as avoidance, following, and approaching. It incorporates a set of social metrics, including proxemic invasion rate, detour distance, and interaction comfort, to evaluate the social acceptability of robotic navigation strategies. The platform supports testing of different local planners and provides a structured environment for benchmarking socially aware navigation algorithms.

Choi et al. [58] developed a shopping-assistant robot navigation system using an Enhanced Potential Field (EPF) method to improve human–robot coexistence. Implemented on an ROS and Gazebo platform, the system dynamically adjusted repulsive forces based on pedestrian proximity to produce smoother and more socially compliant avoidance behaviours. Simulation experiments in a virtual supermarket environment demonstrated reduced path oscillation and fewer abrupt turns compared to conventional potential field methods, indicating improved trajectory efficiency and potential for human comfort.

In addition, ongoing studies are exploring the integration of semantic SLAM with affective recognition, enabling robots not only to “see” the position of humans but also to infer their intentions and emotional states. Arce et al. [59] implemented a multimodal emotion recognition system for human–robot interaction in industrial-style environments. The system fuses visual, auditory, and skeletal data to classify emotional states, such as happiness, frustration, or stress, using a hybrid sensor suite. These emotional cues are mapped to collaborative control parameters—such as robot speed, spacing, and task timing—enabling adaptive behavioural responses. Evaluated in laboratory setups using real human participants, the system achieved reliable emotion classification and demonstrated enhanced interaction fluidity, highlighting the potential of emotion-aware adaptation in future industrial robotics.

The shift from “collision avoidance” to “social norm compliance” is a critical prerequisite for the deployment of robots in public spaces. Future research directions will focus on the real-time recognition of multimodal interactive cues, the dynamic modelling of social behaviours, and the cross-cultural adaptation of social norms [60].

### 5.4. The Future of ROS 2 in Industrial Applications

Building upon the architectural advantages of ROS 2, as systematically reviewed in Section 2, particularly its innovations in communication mechanisms, modular decoupling, and multi-platform compatibility, ROS 2 is increasingly demonstrating substantial potential for deployment in industrial scenarios. In this section, practical application cases are further explored to analyse the adoption of ROS 2 in industrial environments and to discuss its future development trends.

With the integration of DDS (Data Distribution Service) middleware, the ROS 2 platform supports real-time scheduling and multi-platform deployment, marking a significant advancement in reliability, security, and scalability. Compared to ROS 1, ROS 2 provides support for real-time multi-threaded task execution (compatible with real-time operating systems), enhanced security mechanisms such as data encryption and authentication, and robust cross-platform compatibility, including support for Linux, Windows, and macOS, as well as embedded and industrial control systems. These improvements result in significantly enhanced performance in control frequency, communication latency stability, and inter-node communication capability, thereby addressing many of the limitations previously encountered in ROS 1 for industrial-grade applications.

As practical demonstrations of ROS 2’s industrial adaptability, Storiale et al. [61] proposed a robot-agnostic ROS 2 controller architecture that has been successfully deployed on UR10 and Comau robotic arms, demonstrating excellent hardware interoperability. Papavasileiou et al. [62] developed a voice-enabled task coordination framework using Behaviour Trees, facilitating human–robot collaborative inspection in industrial environments. Furthermore, the deep integration of ROS 2 with the Gazebo and Ignition simulation platforms enables digital twin modelling and large-scale deployment testing [18], significantly reducing development costs and enhancing repeatability in industrial systems [63].

In the domain of agricultural automation, Jin et al. [55] leveraged ROS 2 to enable multi-sensor fusion for fruit tree recognition and path generation, significantly improving operational efficiency in horticultural tasks. Similarly, Asiminari et al. [64] demonstrated the effective integration of ROS 2 within agricultural robot systems for route planning and task scheduling, highlighting its potential for seamless collaboration with perception modules such as crop recognition and environmental awareness.

Overall, the evolution of ROS-based navigation systems is characterised by several converging trends: enhanced environmental perception through multi-sensor fusion; real-time adaptability in dynamic scenarios; the incorporation of learning-based planning algorithms; modular and scalable system architectures, such as those supported by ROS 2; and a growing emphasis on safety, robustness, and long-term performance.

These developments collectively pave the way for more intelligent, context-aware, and reliable autonomous navigation across a wide range of real-world applications.

While ROS-based navigation systems are rapidly advancing toward greater intelligence, human–robot interaction, and cloud–edge collaboration, achieving robust and generalizable performance in complex real-world environments remains a significant technical challenge. Key issues, such as the seamless integration of AI models into traditional navigation architectures, reliance on large-scale training datasets, and high computational demands, are yet to be fully addressed. In addition, balancing real-time responsiveness with safety and enabling accurate modelling of human behaviour and compliance with social norms are emerging as critical areas of research. Moving forward, efforts should prioritize the development of transferable, interpretable, and resource-efficient navigation frameworks, alongside the establishment of standardized evaluation benchmarks to support the scalable deployment of ROS systems in both industrial and public domains.

## 6. Conclusions

The findings of this study provide practical and technically informed guidance for engineering practice. Clarifying the functional roles and interface relationships within the ROS navigation stack enables developers to design, configure, and debug robotic systems more effectively.

The comparative analysis of typical obstacle-avoidance algorithms facilitates informed decision-making in algorithm selection based on specific application requirements, thereby enhancing navigation efficiency and safety. Furthermore, the summarised architectural design principles and optimisation strategies are applicable to a wide range of robotic platforms, including service robots and industrial AGVs, contributing to shorter development cycles and improved system performance and reliability.

However, several challenges remain in the domain of ROS-based navigation and obstacle avoidance. First, achieving robust avoidance in highly dynamic and uncertain environments remains difficult. Incorporating advanced prediction models, such as deep learning-based pedestrian intent estimation, can enhance the foresight and reliability of avoidance decisions.

Second, while multi-sensor fusion is already in use, fully leveraging emerging sensors (e.g., mmWave radar and event cameras) and optimising fusion algorithms is essential for improving environmental robustness and perception accuracy. Moreover, to ensure real-time execution on resource-constrained embedded platforms, further research on algorithm lightweighting and hardware–software co-optimisation is necessary.

Lastly, areas such as multi-robot collaborative navigation and planning in human–robot shared spaces require deeper exploration. Establishing standardised benchmarks and open-access datasets will be critical for evaluating the performance of different navigation strategies in complex scenarios and for driving future progress in this field.

## Figures and Tables

**Table 1:** Comparison of key features between ROS 1 and ROS 2.

Comparison Dimension	ROS 1	ROS 2
Communication protocol	Custom TCP/UDP	DDS-based, configurable QoS
System architecture	Centralised (requires roscore)	Decentralised discovery
Platform support	Linux only	Linux, Windows, macOS supported
Nodes and processes	One node per process	Multiple nodes per process supported
Thread model	Fixed callback mechanism	Flexible executors
Lifecycle management	Not supported	Supported via lifecycle nodes
Embedded system support	Limited (via rosserial)	Full support (via micro-ROS)
Parameter system	Runtime access via XMLRPC	Compile-time typing, service-based access
Security mechanism	Requires extension (e.g., SROS)	Built-in DDS security mechanisms
Multi-robot support	Requires multi-master setup	Native support via namespaces and DDS isolation

**Table 2:** Comparison of classical and learning-based local planning methods in robotic navigation.

Method	Planning Paradigm	Typical Scenario	Core Advantages	Main Limitations	Future Improvements	ROS Integration
DWA	Heuristic-based velocity sampling	Static or mildly dynamic environments	High real-time performance; easy ROS integration	Lacks accurate prediction of dynamic obstacles	Behaviour modelling; semantic understanding	Default in move_base
TEB	Graph-based trajectory optimisation	Constrained or narrow spaces	Trajectory smoothness; kinematic feasibility	Depends on initial path; global planner reliance	Robust optimisation; multi-sensor fusion	Supported via teb_local_planner package
MPPI	Stochastic sampling-based control	Highly dynamic and nonlinear environments	Handles complex dynamics; adaptive control	High computational demand; real-time sensitive	Parallelised sampling; variance reduction	Experimental via custom nodes
Reinforcement Learning	Policy learning via trial and error	Unknown or dynamic environments.	Adaptive; generalizable across tasks.	Requires large training data; poor transferability.	Integrated via Gym/Gazebo	hybrid with DWA
Vision-based planning	Semantic image-based perception (CNN, Transformer)	Unmapped, cluttered or low-sensor scenarios.	Scene understanding; reduced map dependency.	Sensitive to lighting, occlusion; limited depth cues.	Implemented in Arena-Rosnav	YOLO plugins

**Table 3:** Quantitative comparison of classical and learning-based local planners in robotic navigation.

Method	Latency	Success Rate	Environment Suitability	Computation Time	References
DWA	20–50 ms control cycle	95% (static), 72.5% (dynamic)	Static or mildly dynamic; obstacle speed ≤ 0.5 m/s.	<10 ms per sample; CPU load <30%	[1,2,3,10,32]
TEB	80–150 ms per optimization cycle	+21.05% (with EKF-based prediction)	Narrow or constrained spaces; turning angle < 60°.	100–200 ms per cycle on i5/i7 CPUs	[8,10,23,24,25]
MPPI	>200 ms per cycle (1000–2000 samples)	92–96% in dynamic dense scenes	High-speed, nonlinear, unstructured environments.	300–500 ms (CPU); <150 ms with GPU	[36,37,38,39]
Reinforcement Learning	150–250 ms (policy inference)	87–94% in known scenarios; less generalizable	Dynamic/uncertain environments.	Varies; real time on GPU/Jetson possible	[31,34]
Vision-based planning	100–200 ms per frame (YOLOv3 Tiny)	90% in good lighting; lower in occlusion	Unstructured or unmapped environments.	120 ms/frame on embedded devices	[35]

## Data Availability

The paper did not generate new data.

## Appendix A

**Table A1:** Open-source implementations and availability of selected methods discussed in this study.

Method/Framework	Description	Link or Availability
Arena-Rosnav	A deep Reinforcement Learning-based navigation framework integrated with ROS and Gazebo simulation environments.	https://github.com/Arena-Rosnav (accessed on 15 March 2025)
FogROS2	A cloud–edge collaborative framework for ROS 2, supporting task offloading and SLAM acceleration.	https://github.com/Arena-Rosnav (accessed on 15 March 2025)
RL-DOVS	A Reinforcement Learning-based extension of the Dynamic Object Velocity Space (DOVS) model for dynamic obstacle avoidance.	Source code not publicly available; readers are advised to contact the authors [27].
YOLO-RRT	A hybrid approach combining YOLO object detection with RRT path planning and obstacle avoidance in Webots-based simulation.	https://github.com/Arena-Rosnav (accessed on 17 March 2025)
